# Aberrant myelomonocytic CD56 expression predicts response to cyclosporine therapy in pediatric patients with moderate aplastic anemia

**DOI:** 10.3389/fped.2023.1272593

**Published:** 2023-12-12

**Authors:** Shanshan Qi, Yu Du, Ming Sun, Lin Zhang, Zhi Chen, Hao Xiong

**Affiliations:** ^1^ Laboratory of Pediatric Hematology, Wuhan Children’s Hospital, Tongji Medical College, Huazhong University of Science and Technology, Wuhan, Hubei, China; ^2^ Department of Hematology, Wuhan Children’s Hospital, Tongji Medical College, Huazhong University of Science and Technology, Wuhan, China

**Keywords:** CD56^+^ myelomonocytes, cyclosporine, moderate aplastic anemia, pediatric aplastic anemia, response

## Abstract

**Objects:**

This study aimed to investigate the expression patterns and clinical significance of neural cell adhesion molecule-positive (CD56^+^) myelomonocytes in pediatric patients with moderate aplastic anemia (mAA).

**Methods:**

Fifty-six pediatric patients with mAA were enrolled in this study. The patients' clinical characteristics, laboratory data, and response to cyclosporine therapy were obtained. CD56 expression on bone marrow myelomonocytic cells was investigated using flow cytometry. The association between aberrant CD56 expression and cyclosporine response was evaluated by a multivariate analysis.

**Results:**

CD56^+^ myelomonocytes were detected in 43% of the mAA cases. Aberrant CD56 expression was frequent on immature CD45^dim^CD16^dim^ granulocytes and mature CD45^bright^CD14^bright^ monocytes. Compared with patients with CD56^−^ myelomonocytes (CD56^−^ patients), patients with CD56^+^ myelomonocytes (CD56^+^ patients) were in moderate hematological condition and had a distinct bone marrow cellular composition profile, which included an increased proportion of myeloid cells and CD56^bright^ natural killer cells and a reduced proportion of CD4^+^ T cells, CD8^+^ T cells, and B cells. The multivariate analysis determined that CD56^+^ myelomonocytes were a favorable factor for achieving response at 6 months after cyclosporine therapy. There was a trend towards a lower 3-year rate of evolution to severe aplastic anemia or relapse among the CD56^+^ patients (8%) than the CD56^−^ patients (22%).

**Conclusion:**

CD56^+^ patients had an increased myeloid compartment and better prognosis compared with CD56^−^ patients. The findings demonstrated the favorable role of CD56^+^ myelomonocytes in aplastic anemia progression.

## Introduction

Aplastic anemia (AA) is a rare but potentially life-threatening disorder (1). Most AA cases (70%–80%) are acquired and are caused by immune-mediated destruction of hematopoietic stem and progenitor cells (2, 3). The first-line therapy for AA depends on the patient's age and the severity of the disease. There is no standard management for patients with moderate aplastic anemia (mAA). Although mAA is generally recommended for observation alone until progression to severe aplastic anemia (sAA), the results of current studies justify an early intervention with cyclosporine (4, 5). However, the predictive factors for the efficacy of cyclosporine therapy have been less well studied.

Also known as neural cell adhesion molecule (NCAM), CD56 belongs to the immunoglobulin superfamily of cell adhesion molecules. *CD56* mRNA is generated by alternative splicing from a single gene, but only three isoforms (NCAM-120, NCAM-140, NCAM-180) are commonly expressed (6, 7). Within the hematopoietic lineage, CD56 is an important marker that defines a population of natural cytotoxic cells, which include natural killer (NK) and NK-T cells (8). Furthermore, CD56 has been detected on other hematopoietic cells, including myeloid cells and monocytes (9–11). Laboratory and clinical observations suggested the unique role of CD56^+^ myelomonocytes in patients with malignant or autoimmune diseases. CD56 expression has been described in different acute myeloid leukemia (AML) genetic subtypes. Indeed, it is frequently associated with t(8;21) RUNX1-RUNX1T1 translocation, AML subtype generally associated with good prognosis (12), but also in the rare CBFA2T3-GLIS2 positive subtype, related with dismal prognosis (13). Patients with rheumatoid arthritis have increased CD56^+^ monocyte frequencies in the peripheral blood, where anti-TNF treatment reduces CD56^+^ monocyte frequency, and the reduction is associated with a better response (14).

Multiparameter flow cytometry reveals myelomonocytic CD56 expression in patients with AA (15). However, detailed analyses of aberrant CD56 expression in AA are limited. In the present study, we clarified the phenotype and function characteristics of CD56^+^ myelomonocytes and assessed whether CD56^+^ myelomonocytes had a predictive value for the outcomes of pediatric patients with AA.

## Subjects and methods

### Patients

Consecutive patients, diagnosed with mAA between January 2019 and December 2022, were enrolled. Bone marrow aspirate specimens for flow cytometric immunophenotyping were obtained from 56 patients before they received immunosuppressive therapy. Ten healthy pediatric stem cell donors served as normal controls. Ethical approval for this study was obtained from the Medical Ethics Committee of Wuhan Children's Hospital, Tongji Medical College, Huazhong University of Science and Technology.

### Diagnosis of AA

The diagnosis of acquired AA was established according to the published criteria (16). Patients were considered to have mAA if they had ≥2 of the following: peripheral blood neutrophils <1.0 × 10^9 ^/L, platelets <50 × 10^9 ^/L, and reticulocytes <60 × 10^9 ^/L. sAA was defined as bone marrow cellularity <25% and at least two of the following: peripheral blood neutrophils <0.5 × 10^9^/L, platelets <20 × 10^9 ^/L, and reticulocytes <20 × 10^9 ^/L.

### Cyclosporine treatment

The mAA patients received cyclosporine (5–6 mg/kg per day, serum level of 200–300 ng/ml) with or without the addition of granulocyte colony stimulating factor for patients with peripheral blood neutrophils <0.2 × 10^9^^ ^/L.

### Response

The hematologic response to cyclosporine was evaluated at 3 and 6 months after treatment initiation. A complete response (CR) was defined as achieving peripheral blood neutrophils >1.5 × 10^9 ^/L, platelets >100 × 10^9 ^/L, and hemoglobin >100 g/L. A partial response (PR) was defined as achieving peripheral blood neutrophils >1.0 × 10^9 ^/L, platelets >50 × 10^9 ^/L, and hemoglobin >80 g/L. Patients with no PR, no CR, or requirement for blood transfusion were deemed as having no response. Relapse was defined by conversion to no response from PR or CR and/or the requirement of blood product transfusion (17).

### Multiparameter flow cytometry

Bone marrow aspirate specimens were obtained at the time of first diagnosis of AA and before treatment initiation, where 3 × 10^5^ nucleated cells per sample were analyzed. Immunophenotyping was performed by multiparameter flow cytometry (BD FACS Canto II system, BD Biosciences, San Jose, CA, USA). The flow panels included the markers CD2, CD3, CD4, CD5, CD7, CD8, CD10, CD11b, CD13, CD14, CD16, CD19, CD20, CD33, CD34, CD38, CD56, CD64, CD71, CD117, HLA-DR, TCRαβ, TCRγδ, and CD45. CD marker expression of >10% was deemed positive (18). Immature/mature granulocytes and monocytes were identified using a CD45/sideward scatter (SSC) backbone strategy and lineage-defined gating, as previously reported (19, 20). Briefly, granulocytes were identified as SSC^int/high^CD45^dim/bright^CD33^dim^, and monocytes were identified as SSC^int^CD45^dim/bright^CD64^+^. Granulocytes and monocytes were also identified by differentiation antigen CD14 and CD16, respectively. Aberrant expression was determined on the basis of deviation from normal patterns (expression intensity at least 0.5 decades disparate from the corresponding normal cells from healthy pediatric stem cell donors). The percentage of the CD56^+^ population in myelomonocytes was assessed in a graded regimen: +, positive in 10%–30%; ++, positive in 31%–60%; +++, positive in >60%; negative, positive in <10%. The mean fluorescence intensities of CD56^+^ myelomonocytes were recorded.

### Statistical analyses

All calculations were performed using SPSS 20 (IBM, Armonk, NY, USA). Continuous variables with non-normal distribution are expressed as the median and range, and the Mann–Whitney *U*-test was used for statistical analysis. Categorical variables are presented as frequencies and proportions (%) and analyzed by the *χ*^2^ test (or Fisher's exact test for expected cell frequencies <5). The associations between clinical variables with response to cyclosporine were calculated by a multivariate logistic regression analysis. The Kaplan–Meier curve demonstrated the cumulative incidence of the response to cyclosporine and time to event, and the differences were evaluated by the log-rank test. *P* < 0.05 was considered statistically significant.

## Results

### Clinical characteristics of patients

Of the 56 mAA patients enrolled, 29 were male and 27 were female (male:female ratio, 1.07:1). The median age was 4.7 years (range: 0.1–15.7 years). Table 1 presents the patients' characteristics. The median time between diagnosis and treatment was 7 days (range: 0–35 days). At mAA diagnosis, 14 patients (25%) presented with infection and the median C-reactive protein (CRP) level was 2.11 mg/L (range: 0.10–118.9 mg/L). Among the 56 patients, 24 had CD56^+^ myelomonocytes and were classified as CD56^+^ patients. The remaining 32 patients without CD56^+^ myelomonocytes were classified as CD56^−^ patients. There were no significant differences between the CD56^+^ and CD56^−^ patients in the gender and age distributions, duration between diagnosis and treatment, presence of infection, CRP concentration, and platelet count. However, the CD56^+^ patients had higher neutrophil counts and hemoglobin levels than the CD56^−^ patients at the time of diagnosis.

**Table 1:** Clinical characteristics of pediatric mAA patients.

Variable	All patients (*n* = 56)	CD56^−^ patients (*n* = 32)	CD56^+^ patients (*n* = 24)	*P*
Sex, male/female	29/27	15/17	14/10	0.430
Age, years [median (range)]	4.7 (0.1–15.7)	4.2 (0.1–15.7)	5.2 (0.1–14.4)	0.585
Days between diagnosis and treatment [median (range)]	7 (0–35)	7 (0–35)	7 (0–12)	0.863
Presenting symptoms at diagnosis				0.543
Infection	14	9	5	
Others^a^	44	23	21	
CRP, mg/L [median (range)]	2.11 (0.10–118.9)	2.59 (0.10–118.9)	0.78 (0.75–19.55)	0.158
Hematologic parameters in peripheral blood [median (range)]				
Neutrophil count, ×10^9^/L	0.35 (0.02–0.9)	0.31 (0.02–1.33)	0.93 (0.09–4.09)	**0**.**032**
Platelet count, ×10^9^/L	17 (0–87)	15 (0–87)	24 (3–83)	0.194
Hemoglobin, g/L	79 (44–105)	75 (44–103)	90 (70–105)	**<0**.**001**

CRP, C-reactive protein.

^a^ Includes none, anemia and bleeding. Values in bold denote statistical significance at the level *P* < 0.05.

### Flow cytometric immunophenotyping

Analysis of the patients' bone marrow immunophenotype data revealed that all cases had normal expression of lymphocyte subset cell surface markers. The lymphocytic antigens involved in this study included: the T cell markers CD2, CD3, CD4, CD5, CD7, and CD8; B cell markers CD19 and CD20; and the NK cell markers CD16 and CD56. Immature/mature granulocytes and monocytes exhibited normal myelomonocytic markers: CD4, CD11b, CD13, CD14, CD16, CD33, CD64, and HLA-DR.

**Figure 1:**
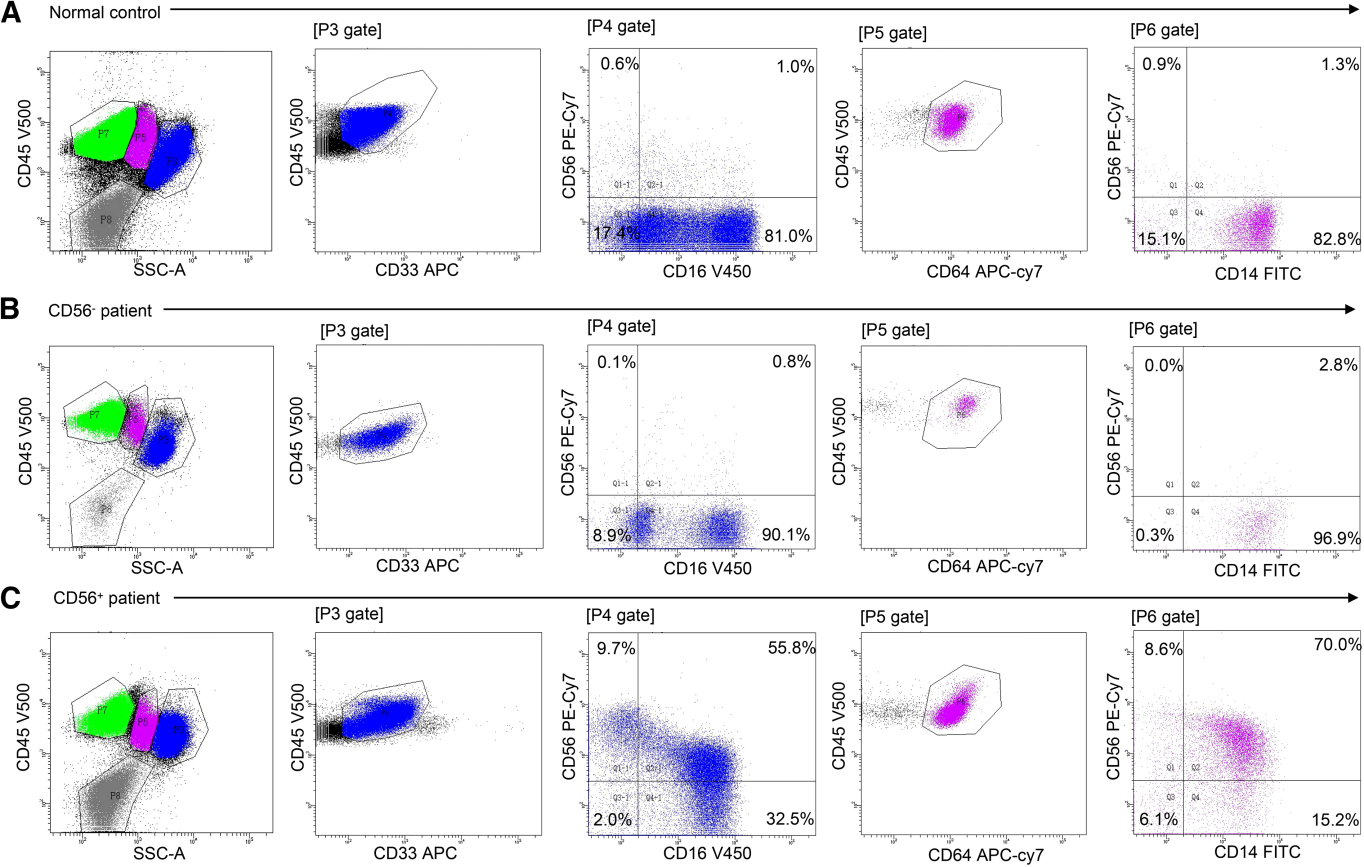
Flow cytometric analysis of myelomonocytic CD56 expression in normal control, CD56^−^ patients, and CD56^+^ patients. Cell populations were identified using CD45 expression and sideward scatter (SSC). Granulocytes were defined as SSC^int/high^/CD45^dim/bright^/CD33^dim^ cells. Monocytes were defined as SSC^int^/CD45^dim/bright^/CD64^+^ cells. CD16/CD56 and CD14/CD56 dot plots demonstrate aberrant CD56 expression on granulocytes and monocytes, respectively. (**A**) Normal CD56 expression in healthy control bone marrow. (**B**) Normal CD56 expression in mAA. (**C**) Aberrant CD56 expression on granulocytes and monocytes in mAA.

**Figure 2:**
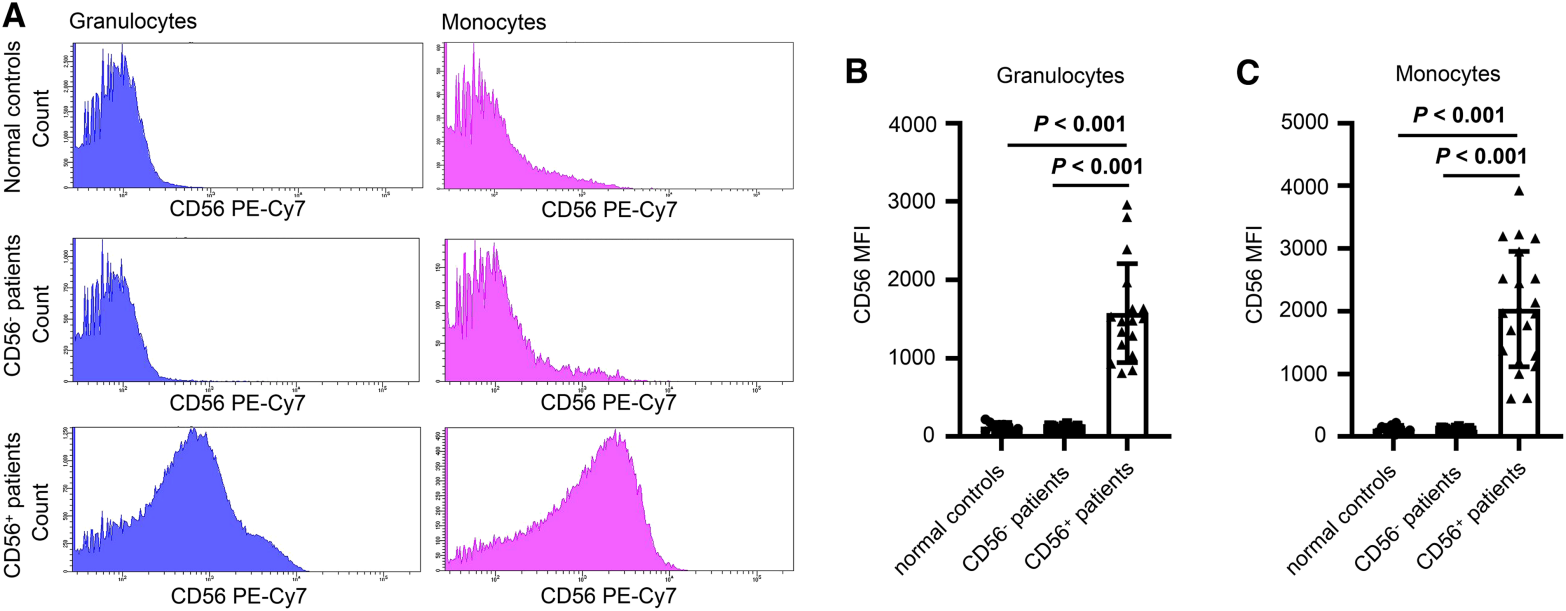
(**A**) Representative fluorescence spectrum showing CD56 expression in normal controls, CD56^−^ patients and CD56^+^ patients. (B and C) Mean fluorescence intensity (MFI) of granulocytes (**B**) and monocytes (**C**) of normal controls, CD56^−^ patients, and CD56^+^ patients. Values in bold denote statistical significance at the level *P* < 0.001.

Twenty-four patients (24/56, 43%) had CD56^+^ myelomonocytes [three cases (13%) with CD56^+^ granulocytes, seven cases (29%) with CD56^+^ monocytes, and 14 cases (58%) with both CD56^+^ granulocytes and CD56^+^ monocytes]. CD56 was expressed frequently on CD16^dim^CD33^dim^ immature granulocytes (15/24, 62.5%) and CD14^bright^CD64^+^ mature monocytes (18/24, 75%). Aberrant CD56 expression was also observed on CD16^bright^CD33^dim^ mature granulocytes (5/24, 20.8%) and CD14^dim^CD64^+^ immature monocytes (3/24, 12.5%), but were infrequent. Table 2 details the immunophenotyping results. Figure 1 depicts representative CD56 dot plot distributions of the CD56^+^ and CD56^−^ patients on flow cytometry. Figure 2 shows the profile of mean fluorescence intensity of CD56^+^ myelomonocytes.

**Table 2:** Granulocyte and monocyte immunophenotypes in CD56^+^ patients.

	CD56^+^ granulocytes (*n* = 17)		CD56^+^ monocytes (*n* = 21)	
Case	Positive subset	Immunophenotype	Positive subset	Immunophenotype
1	+	CD45^dim^CD16^dim^	+	CD45^bright^CD14^bright^
2	++	CD45^dim^CD16^dim^/CD45^bright^CD16^bright^	+++	CD45^dim^CD14^dim^
3	++	CD45^bright^CD16^bright^	Neg	/
4	+++	CD45^bright^CD16^bright^	+++	CD45^bright^CD14^bright^
5	+	CD45^dim^CD16^dim^	Neg	/
6	+	CD45^dim^CD16^dim^/CD45^bright^CD16^bright^	++	CD45^bright^CD14^bright^
7	Neg	/	+	CD45^bright^CD14^bright^
8	+++	CD45^dim^CD16^dim^	+++	CD45^bright^CD14^bright^
9	+++	CD45^dim^CD16^dim^/CD45^bright^CD16^bright^	+++	CD45^bright^CD14^bright^
10	+	CD45^dim^CD16^dim^	+	CD45^bright^CD14^bright^
11	Neg	/	+	CD45^bright^CD14^bright^
12	Neg	/	++	CD45^dim^CD14^dim^
13	Neg	/	+	CD45^bright^CD14^bright^
14	Neg	/	++	CD45^bright^CD14^bright^
15	+	CD45^dim^CD16^dim^	+	CD45^bright^CD14^bright^
16	++	CD45^dim^CD16^dim^	++	CD45^bright^CD14^bright^
17	+++	CD45^dim^CD16^dim^	Neg	/
18	++	CD45^dim^CD16^dim^	++	CD45^bright^CD14^bright^
19	Neg	/	++	CD45^bright^CD14^bright^
20	++	CD45^dim^CD16^dim^	+++	CD45^bright^CD14^bright^
21	+++	CD45^dim^CD16^dim^	+++	CD45^bright^CD14^bright^
22	Neg	/	+	CD45^bright^CD14^bright^
23	++	CD45^dim^CD16^dim^	+	CD45^bright^CD14^bright^
24	+++	CD45^dim^CD16^dim^	++	CD45^dim^CD14^dim^

+, positive in 10%–30% of cells; ++, positive in 31%–60% of cells; +++, positive in >60% of cells; neg, negative: positive in <10% of cells.

### Bone marrow cellular composition

**Figure 3:**
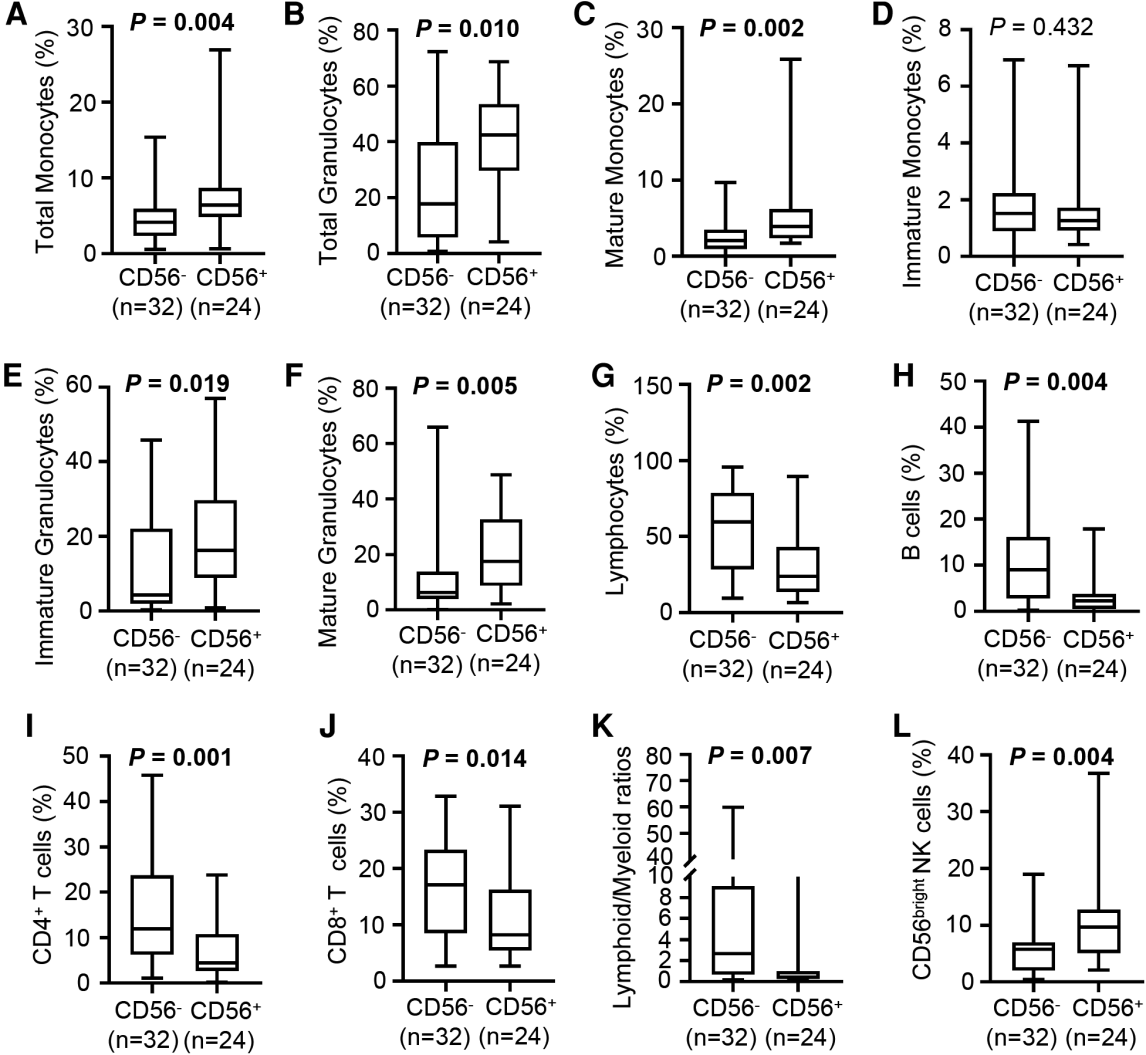
Cellular composition of bone marrow in CD56^−^ and CD56^+^ patients. (A–F) Box plots depict the percentage of total monocytes (**A**), total granulocytes (**B**), mature monocytes (**C**), immature monocytes (**D**), immature granulocytes (**E**), and mature granulocytes (**F**) in CD56^−^ and CD56^+^ patients. (G–J) Box plots depict the percentage of lymphocytes (**G**), B cells (**H**), CD4^+^ T cells (**I**), and CD8^+^ T cells (**J**) in CD56^−^ and CD56^+^ patients. (**K**) Box plot depicts the lymphoid/myeloid ratios in CD56^−^ and CD56^+^ patients. (**L**) Box plot depicts the percentage of CD56^bright^ NK cells in CD56^−^ and CD56^+^ patients. Lines in the boxes indicate medians; bottom and top whiskers indicate minimum and maximum values, respectively. The cell population percentage represented the proportion within all nucleated cells. Values in bold denote statistical significance at the level *P* < 0.05.

The proportions of monocytes, granulocytes, and lymphocytes in nucleated cells were assessed by flow cytometry. Compared with the CD56^−^ patients, the CD56^+^ patients had increased percentages of monocytes (median, 6.5% vs. 4.1%) and granulocytes (median: 42.4% vs. 17.8%; Figures 3A,B) and a higher proportion of mature monocytes (Figures 3C,D). The CD56^+^ patients had a higher percentage of immature granulocytes than the CD56^−^ patients (median: 16.2% vs. 4.3%) (Figure 3E).

Paralleling the increased immature granulocytes, the CD56^+^ patients had an increased percentage of mature granulocytes compared with the CD56^−^ patients (median: 17.6% vs. 6.3%; Figure 3F). Likely partly due to the increased myeloid cells, the CD56^+^ patients had a strongly reduced proportion of lymphocytes compared with the CD56^−^ patients (median: 23.9% vs. 59.6%), which included reduced B cells, CD4^+^ T cells, and CD8^+^ T cells (Figures 3G–J). Unsurprisingly, the CD56^+^ patients had a lower lymphoid/myeloid ratio than the CD56^−^ patients (median: 0.4 vs. 2.7) (Figure 3K). Interestingly, the CD56^+^ patients had an increased percentage of CD56^bright^ NK cells compared with the CD56^−^ patients (median: 9.7% vs. 5.8%, Figure 3L).

### Treatment and outcomes

**Figure 4:**
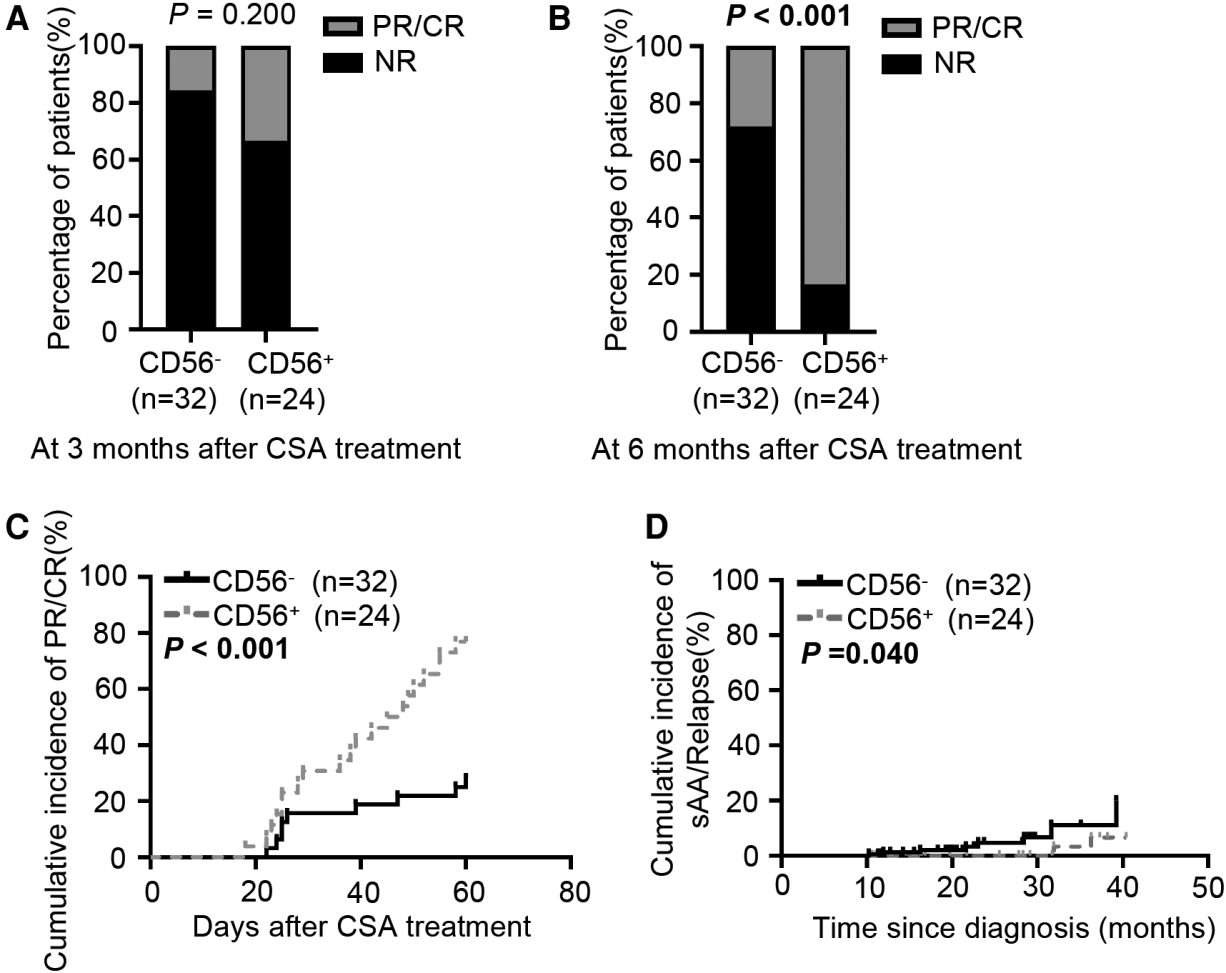
Response to cyclosporine therapy in CD56^−^ and CD56^+^ patients. (A, B) Bar graphs depict the response rate to cyclosporine treatment at 3 (**A**) and 6 months (**B**) (**C**) Cumulative PR or CR incidence after cyclosporine treatment. (**D**) Cumulative incidence of sAA or relapse after cyclosporine treatment. CSA, cyclosporine. Values in bold denote statistical significance at the level *P* < 0.05.

In China, cyclosporine is more frequently used for pediatric AA as it costs less and produces fewer adverse effects than anti-thymocyte globulin (21, 22). We evaluated the hematologic response of pediatric mAA patients to cyclosporine therapy at 3 and 6 months after treatment initiation. The 3-month response rate to cyclosporine did not differ significantly between the two groups (33% vs. 16%, *P* = 0.200, Figure 4A). At 6 months, 83% of the CD56^+^ patients achieved PR or CR. However, only 28% of the CD56^−^ patients responded to cyclosporine (*P* < 0.001, Figure 4B). Kaplan-Meier analysis demonstrated that the cumulative response rate of the CD56^+^ patients was significantly higher than that of the CD56^−^ patients (*P* < 0.001, Figure 4C). We also found that the cumulative response rates had no difference between patients with CD56^+^ mature monocytes and CD56^+^ immature monocytes (Supplementary Figure S1). Multivariate analysis of laboratory variables at the time of diagnosis revealed that age (older or younger than 4.7 years), sex (male or female), presence or absence of infection, peripheral platelet (more or less than 20 × 10^3 ^/µl), and reticulocyte count (more or less than 20 × 10^3^/µl) could not be deemed as predictors. Only two parameters (peripheral neutrophils >500^ ^/µl and abnormal CD56 expression) were significant factors associated with good response to cyclosporine therapy at 6 months (Table 3). Subsequently, we analyzed the patients' prognoses after cyclosporine therapy. The median follow-up time was 20.5 months (range: 10.2–40.4 months). In contrast to the response rate, the probability of evolution into sAA or relapse at 3 years tended to be higher in CD56^−^ patients (22%) than in CD56^+^ patients (8%) (Figure 4D).

**Table 3:** Multivariate analysis of predictive factors for response to cyclosporine therapy at 6 months.

Variable	Risk ratio	95% confidence interval	*P*
Age at diagnosis, years: >4.7 vs. ≤4.7	1.24	0.30–5.14	0.768
Sex: male vs. female	1.56	0.39–6.44	0.539
Presenting symptoms: Infection vs. other	3.38	0.68–16.70	0.136
Neutrophils (/µl): ≥500 vs. <500	0.22	0.05–0.96	**0**.**043**
Platelets (×10^3^/µl): ≥20 vs. <20	1.36	0.33–5.61	0.674
Reticulocytes (10^3^/µl): ≥20 vs. <20	0.43	0.11–1.73	0.235
CD56^+^ vs. CD56^−^	0.21	0.05–0.93	**0**.**040**

Values in bold denote statistical significance at the level *P* < 0.05.

## Discussion

In this study, we evaluated the bone marrow cellular component in CD56^+^ or CD56^−^ mAA patients and the relationship between myelomonocytic CD56 expression and the response to cyclosporine therapy. We determined that the CD56^+^ patients had less severe myeloid cell reduction in comparison with the CD56^−^ patients. Furthermore, we detected a better response to cyclosporine among the CD56^+^ patients than the CD56^−^ patients.

To date, the functional role of CD56^+^ immune cells is not fully understood, but CD56 expression has been hypothesized to promote immune cell proliferation and differentiation (23, 24). A proposed mechanism for the functional role of CD56 depends on its binding to a number of proteins or extracellular matrix molecules. In particular, the homophilic interaction between CD56 molecules can induce the preferential activation and expansion of CD56^+^ immune cells (25, 26). Additionally, upregulated CD56 expression is strongly associated with activation of the MAPK signaling pathway, leading to increased glycolysis and resistance to apoptosis (27, 28). These pathophysiological mechanisms may explain the possible reason the CD56^+^ patients had better hematological conditions than the CD56^−^ patients.

Several studies highlighted the immune regulation imbalance in patients with acquired AA (29–32). Immune dysfunction occurs mainly due to the cellular hyperimmune state and leads to a corresponding immune tolerance disorder, where the overactivated immune cells in such patients are unable to recognize auto-hematopoietic cells. CD8^+^ T lymphocytes are the main effector cells in the immune system. CD8^+^ T cell clonal amplification and overactivation inhibits bone marrow hematopoietic functions and induces hematopoietic cell apoptosis (31, 33). NK cells are crucial in AA pathogenesis (29). Specifically, CD56^bright^ NK cells inhibit T cell immunity overactivation and subsequently reduce the T cell-induced damage of hematopoietic stem and progenitor cells in AA (34, 35). In the present study, the CD56^+^ patients demonstrated a strong decrease in CD8^+^ T cells and a significant increase in CD56^bright^ NK cells compared with the CD56^−^ patients. The homophilic interaction function of CD56 might explain the low percentage of cytotoxic lymphocytes in CD56^+^ patients. CD56 was reported to cooperate with leukocyte function-associated antigen-1 and -3 to enhance interactions between CD56^+^ immune cells, leading to CD56-mediated lysis (36, 37). As a consequence, we speculate that CD56^+^ populations might reshape the immune balance of the bone marrow microenvironment and be responsible for the higher response rate to cyclosporine therapy in mAA patients.

Several limitations in our study merit consideration. First, only 24 CD56^+^ patients were enrolled due to the small patient cohort. Second, sAA patients were not enrolled in this study due to the severe bone marrow hypocellularity. Testing for myelomonocytic CD56 expression at diagnosis had poor sensitivity for sAA. Finally, this study was comparatively preliminary, and the exact pathological mechanism of CD56^+^ myelomonocytes in pediatric patients with acquired AA remains unclear.

In summary, we demonstrated that CD56^+^ myelomonocytes accompanied an increased percentage of myeloid cells in the bone marrow of mAA patients, which suggested the remodeling effect of myelomonocytic CD56 expression on bone marrow cellular components. Importantly, we determined that myelomonocytic CD56 expression was associated with a better response to cyclosporine therapy. Additional studies with a larger patient cohort are needed to validate myelomonocytic CD56 expression as a response predictor in patients with acquired AA.

## Data Availability

The raw data supporting the conclusions of this article will be made available by the authors, without undue reservation.
