# Supplementary material for: Aberrant myelomonocytic CD56 expression predicts response to cyclosporine therapy in pediatric patients with moderate aplastic anemia
Source: Front Pediatr. 2023 Dec 12;11:1272593. doi: 10.3389/fped.2023.1272593 (PMC10751928; doi:10.3389/fped.2023.1272593)
Supplement: Supplementary file 1 [file Image1.pdf]

Supplemental Figure and Figure legend

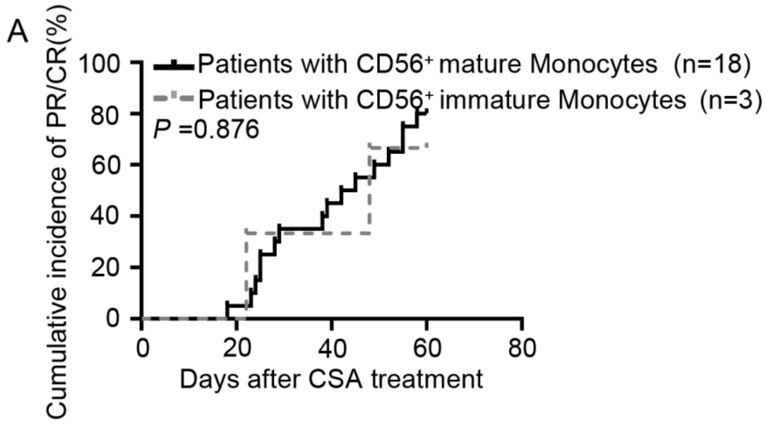

Supplemental Figure 1. (A) Cumulative PR or CR incidence of patients with CD56^{+} mature monocytes and CD56^{+} immature monocytes after cyclosporine treatment (*P* =0.876).
